# Late Manifestation of Massive Jejunal and Cecal Varices Post Liver and Small Bowel Transplantation in a Patient With Microvillus Inclusion Disease

**DOI:** 10.7759/cureus.15884

**Published:** 2021-06-24

**Authors:** Shiva F Naidoo, Joshua C Obuch

**Affiliations:** 1 Internal Medicine, Geisinger Health System, Wilkes-Barre, USA; 2 Gastroenterology, Geisinger Health System, Wilkes-Barre, USA

**Keywords:** gastric varices, acute gastrointestinal bleed, microvillus inclusion disease, jejunal varices, small bowel transplant, liver transplant, upper gastro-intestinal bleed

## Abstract

We report the case of an 18-year-old male with a medical history of microvillous inclusion disease (MID) and notable surgical history of small bowel, liver, and pancreas transplant who presented with massive jejunal and cecal varices. Endoscopy findings demonstrated a large grape-like cluster, with subsequent CT angiography (CTA) showing other variceal lesions in the cecum. The patient was transferred to the original transplant center for recommended open surgical evaluation and combined interventional radiology (IR) embolization of varices. MID is a rare genetic disorder caused by mutations in the Myosin VB (MYO5B) gene leading to a lack of myosin Vb. Patients subsequently develop liver damage at birth, which necessitates a small bowel/liver transplant in childhood.

## Introduction

Microvillus inclusion disease (MID) is a rare genetic disorder due to mutations in the Myosin VB (MYO5B) gene resulting in a lack of myosin Vb. Patients who suffer from this condition cannot form microvilli and are total parenteral nutrition (TPN)-dependent from birth, which can lead to sequelae of liver damage. Treatment for patients affected by MID includes small bowel transplant and possible liver transplant in the setting of associated liver damage [1]. Jejunal varices are a rare cause of gastrointestinal (GI) bleeding with associated high mortality [2] and most often occur in the setting of portal hypertension. In this report, we discuss the case of an 18-year-old male with MID post small bowel and liver transplant in infancy who presented with recurrent GI bleeding as a consequence of jejunal varices.

## Case presentation

An 18-year-old male with a past medical history of MID and a notable surgical history of small bowel, liver, and pancreas transplant, who was maintained on tacrolimus 2 mg and prophylactic trimethoprim-sulfamethoxazole 400-80 mg, presented to the emergency room (ER) with four days of hematochezia and lightheadedness. He had experienced recurrent GI bleeding, most recently a year ago, and had undergone multiple endoscopies by pediatric gastroenterology in the past, which had been negative for active bleeding or any possible source. The patient denied nausea, vomiting, abdominal pain, fevers, shortness of breath, cough, or chest pain. His vitals on presentation were as follows: BP of 136/67 mmHg, heart rate (HR) of 114 beats per minute, respiratory rate (RR) of 22 breaths per minute, O_2_ saturation of 96% on room air, and temperature of 36.9 °C.

**Figure 1:**
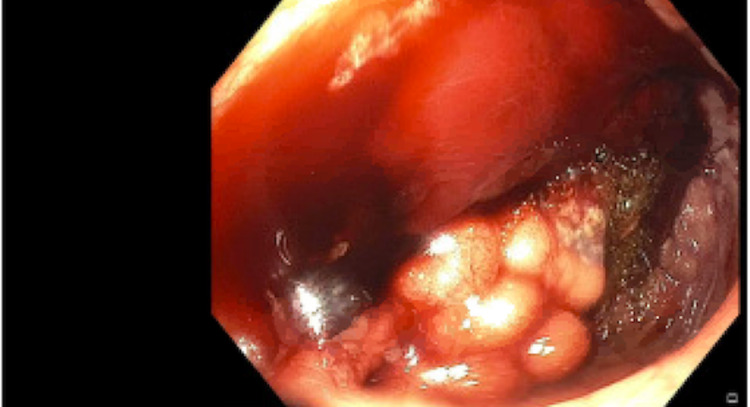
Hemorrhagic mucosa in proximal jejunum with varices

**Figure 2:**
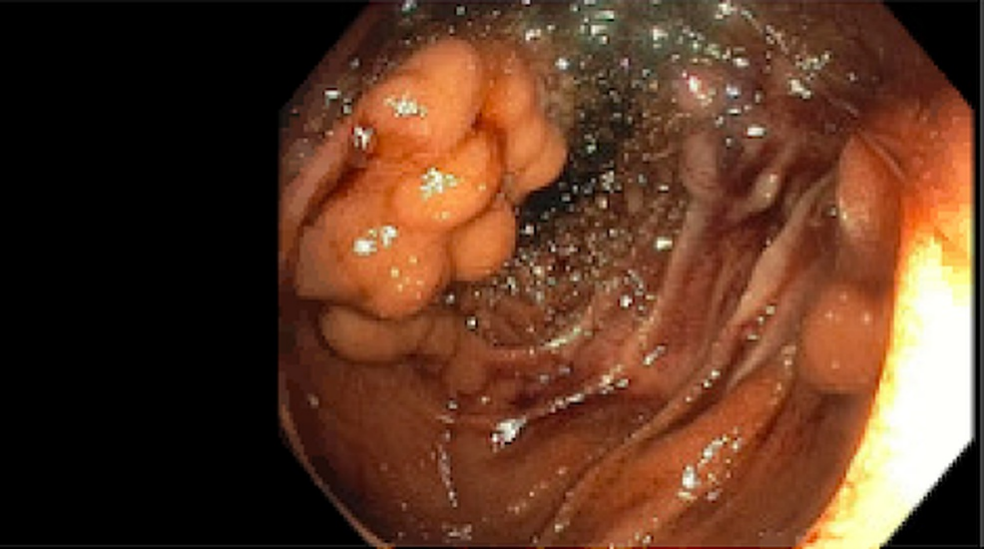
Post-bleeding biopsy with hemostasis achieved

**Figure 3:**
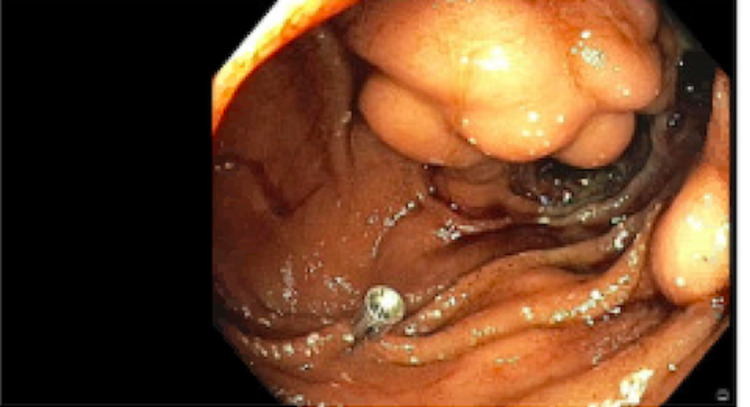
Metallic clip placed with hemostasis maintained

The serologic evaluation was pertinent for hemoglobin of 9.6 g/dL, white blood cell count of 4.66 K/uL, platelets of 130 K/uL, and prothrombin time (PT)-international normalized ratio (INR) of 1.152. Liver function tests including aspartate aminotransferase (AST), alanine aminotransferase (ALT), alkaline phosphatase (ALP), and bilirubin were all within normal limits. The patient was admitted and scheduled to undergo evaluation with esophagogastroduodenoscopy (EGD). The following day, he continued to deteriorate with ongoing bleeding resulting in a hemoglobin drop to 6.7 g/dL, requiring transfusion of one unit of packed red blood cells. EGD with push enteroscopy showed an enteroenterostomy with a hemorrhagic appearance in the jejunum. The area adjacent to the anastomosis was biopsied to rule out rejection. The region near the anastomosis had grape-like clustering raising concern for varices, and noted hemorrhage post-biopsy (Figure 1). Hemostasis was achieved with argon plasma coagulation and epinephrine (Figure 2). A metallic clip was placed for radiographic identification for the purpose of an interventional radiology (IR) procedure (Figure 3). Subsequently, the patient remained intubated and was transferred to ICU on an octreotide drip.

**Figure 4:**
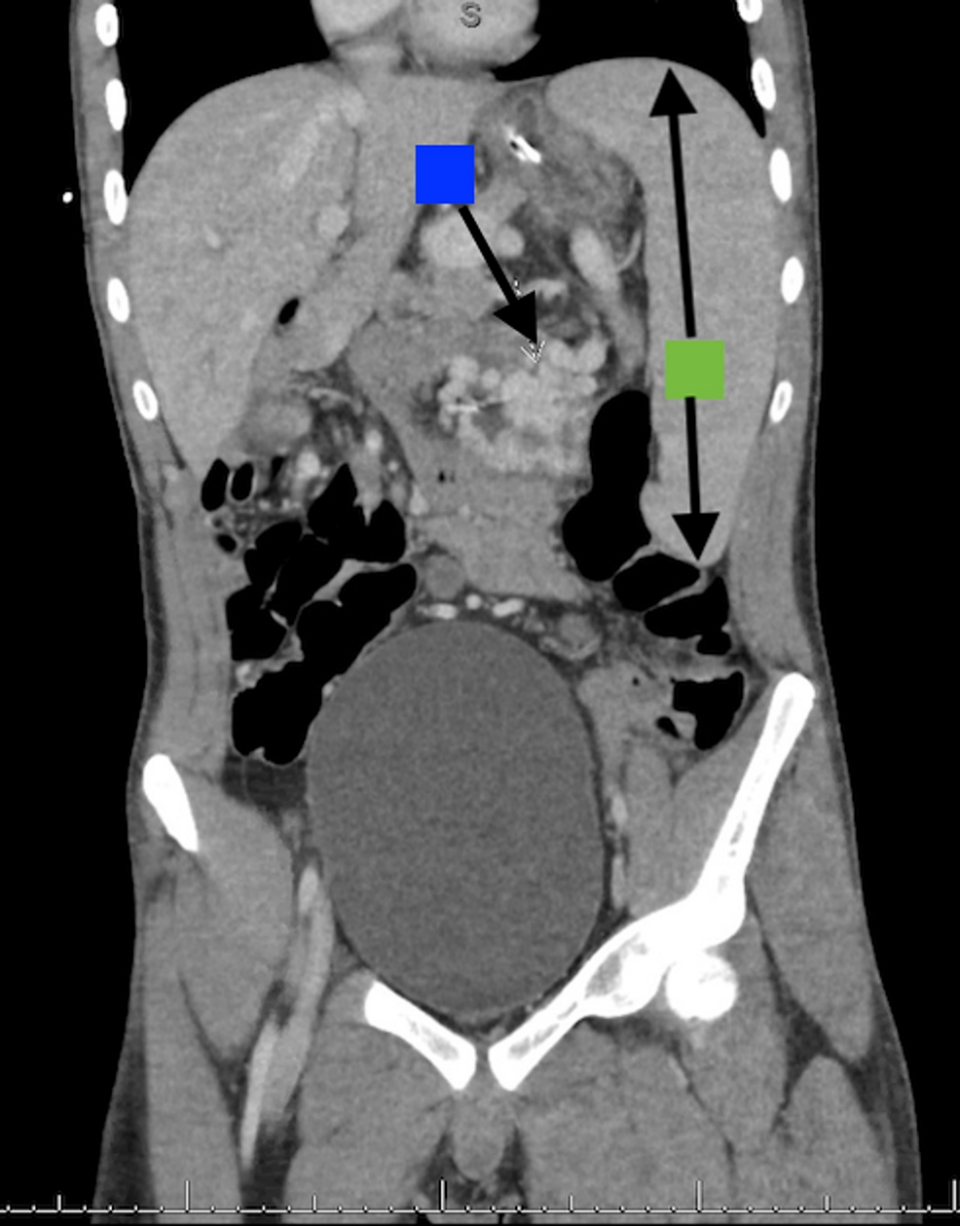
CT angiogram of the abdomen showing massive jejunal varices (blue arrow), and massive splenomegaly (green bidirectional arrow) of a craniocaudal length of 20 cm CT: computed tomography

**Figure 5:**
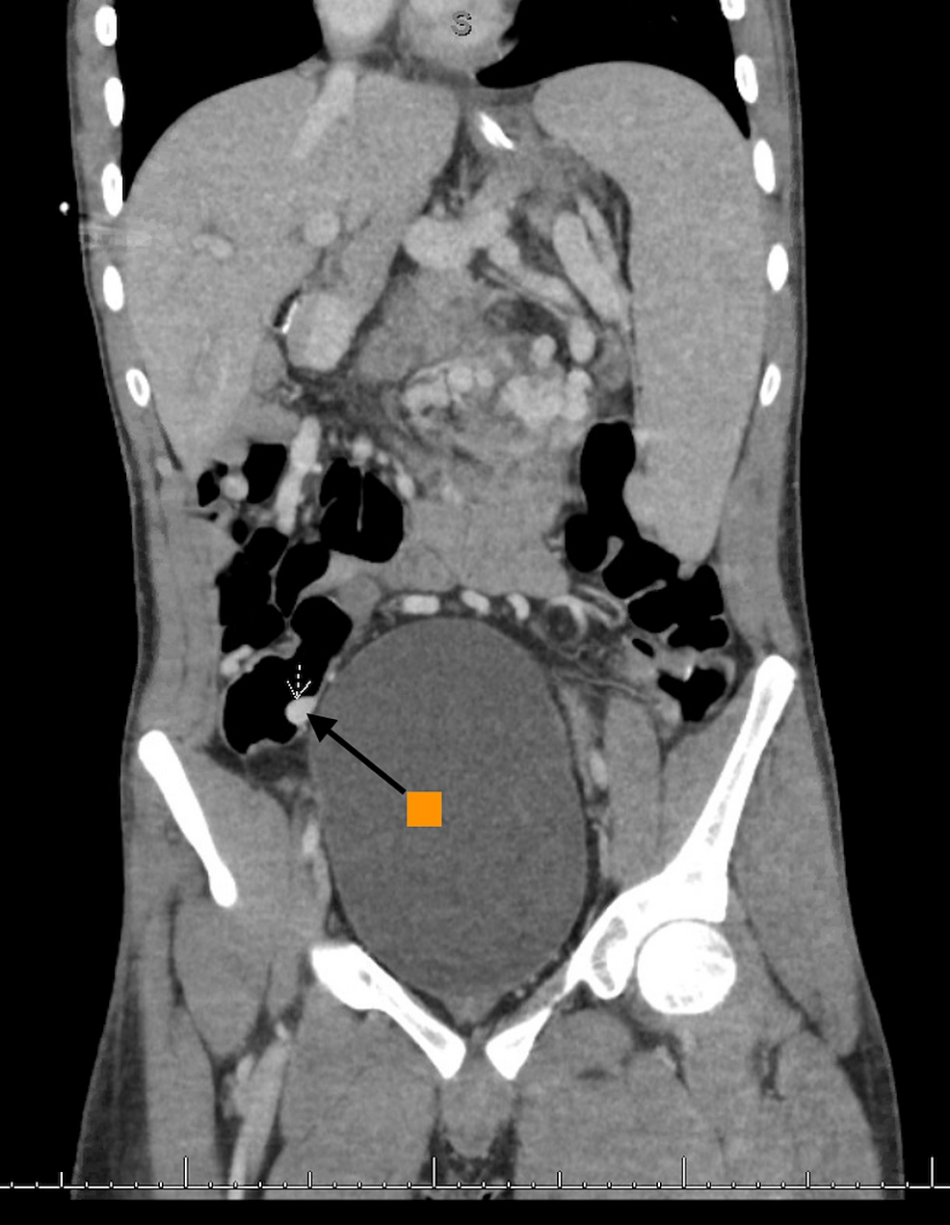
CT angiogram of the abdomen showing small second cluster of cecal varices (orange arrow) CT: computed tomography

The case was discussed with IR and Transplant Hepatology, as endoscopic management with sclerosing agents or glue was not available. The complex nature of the post-surgical vascular anatomy and concern for an interrupted inferior vena cava prompted IR to perform a CT angiography (CTA) of the abdomen, with the possibility of embolization. The results of the CTA demonstrated a massive tangle of jejunal submucosal varices, as well as reported cecal submucosal varices and massive splenomegaly (Figures 4, 5). Furthermore, due to atrophic hepatic vein and systemic collaterals to the azygos vein, transjugular intrahepatic portosystemic shunting (TIPS) was deemed not feasible at the time. Subsequent discussion involving Transplant Surgery at the hospital where the patient had received his small bowel and liver transplant prompted a decision to transfer the patient for open surgical evaluation and combined IR embolization of varices. The patient was then stabilized and transferred.

## Discussion

Ectopic varices contribute to 2-5% of all GI bleeding [2]. Concurrently, there is also a four-fold higher risk of bleeding when compared to esophageal varices, with a mortality rate as high as 40% [3]. The causes of ectopic varices are varied, and patients with portal hypertension are at increased risk for developing them. In a systematic review of 169 patients with bleeding from ectopic varices, only 18% of intraabdominal bleeds were found to have originated at the jejunum and 7-12% demonstrated jejunal stomal or parastomal bleeds [4]. Also, prior abdominal surgery is a predisposing factor that can lead to jejunal varices [5]. Small-bowel varices can result from mesenteric hypertension, mediated by portal hypertension or mesenteric vein stenosis [6]. At the anastomotic site of the enteroenterostomy, small-bowel varices can occur, as demonstrated in this case. A complication of portal hypertension can present as jejunal varices and, in rare cases, cecal varices. A similar case report by Ayash et al. [7] has documented portal venous thrombosis from a Factor V Leiden deficiency manifesting as another cause of jejunal varices.

Unlike most cases, our patient had a unique and rare anatomic predisposition. The hepatic vein was atrophic, along with azygos collaterals to the superior vena cava that greatly contributed to the presence of portal hypertension. Generally, portal hypertension would affect the esophageal or gastric vessels [8]. However, the patient developed jejunal and cecal varies, despite undergoing multiple endoscopies in the past.

The management of jejunal or ectopic varices can be challenging because of their varying locations and clinical presentations. Specialized techniques generally include surgical, endoscopic, or endovascular approaches. Surgical management may involve laparotomy with ligation of the vessel at the affected site [9]. Further intervention may extend as far as the resection of the location of the small bowel affected as well [10]. Endoscopic management may include obliteration of jejunal varices with the use of cyanoacrylate glue through a double-balloon enteroscopy [11]. Endovascular approaches include balloon-occluded retrograde transvenous obliteration, which is a technique that uses a specialized balloon catheter with a sclerosing agent to stop bleeding [12]. Another common modality employed is percutaneous embolization [13,14]. TIPS is a common treatment modality for ectopic variceal bleeding with portal hypertension, particularly in those associated with a recurrent risk of rebleeding [15]. Portal venous stent placement is a unique approach, in which portal hypertension itself is reduced and subsequent complications including ectopic varices are addressed [16].

## Conclusions

MID is a rare condition, and its treatment includes a small bowel and/or liver transplant. Patients with a history of MID may present with massive jejunal and cecal varices manifesting as GI bleeding, and clinicians should be aware of this phenomenon.
